# Superlubricity of Materials: Progress, Potential, and Challenges

**DOI:** 10.3390/ma16145145

**Published:** 2023-07-21

**Authors:** Maziar Ramezani, Zaidi Mohd Ripin, Cho-Pei Jiang, Tim Pasang

**Affiliations:** 1 Department of Mechanical Engineering, Auckland University of Technology, Auckland 1010, New Zealand; 2 School of Mechanical Engineering, Universiti Sains Malaysia, Nibong Tebal 14300, Malaysia; 3 Department of Mechanical Engineering, National Taipei University of Technology, Taipei 10608, Taiwan; 4 Department of Manufacturing and Mechanical Engineering Technology, Oregon Institute of Technology, Klamath Falls, OR 97601, USA

**Keywords:** friction reduction, nanoscale and macroscale techniques, superlubricity, superlubric materials

## Abstract

This review paper provides a comprehensive overview of the phenomenon of superlubricity, its associated material characteristics, and its potential applications. Superlubricity, the state of near-zero friction between two surfaces, presents significant potential for enhancing the efficiency of mechanical systems, thus attracting significant attention in both academic and industrial realms. We explore the atomic/molecular structures that enable this characteristic and discuss notable superlubric materials, including graphite, diamond-like carbon, and advanced engineering composites. The review further elaborates on the methods of achieving superlubricity at both nanoscale and macroscale levels, highlighting the influence of environmental conditions. We also discuss superlubricity’s applications, ranging from mechanical systems to energy conservation and biomedical applications. Despite the promising potential, the realization of superlubricity is laden with challenges. We address these technical difficulties, specifically those related to achieving and maintaining superlubricity, and the issues encountered in scaling up for industrial applications. The paper also underscores the sustainability concerns associated with superlubricity and proposes potential solutions. We conclude with a discussion of the possible future research directions and the impact of technological innovations in this field. This review thus provides a valuable resource for researchers and industry professionals engaged in the development and application of superlubric materials.

## 1. Introduction

Superlubricity, or the state of near-zero friction between two surfaces, has sparked significant attention in the past decades, underlining its potential to revolutionize various mechanical systems by dramatically enhancing their efficiency. By virtually eliminating friction, superlubricity promises to minimize energy loss, lower maintenance requirements, and extend the operational life of mechanical devices [1,2,3].

This review aims to offer a comprehensive understanding of the superlubricity phenomenon, its principles, the materials exhibiting this characteristic, methods of achieving it, and its potential applications. The paper further outlines the various challenges associated with realizing superlubricity and discusses future research directions that could drive technological advancements in this field.

Superlubricity finds its roots in the early 1990s, with the pioneering work of Hirano and Shinjo [4], who confirmed the existence of superlubricity by measurement of the friction of mica surfaces. They theoretically showed that under specific orientations of crystalline surfaces in contact, the coefficient of friction can approach zero. This was followed by the paper published by Martin et al. [5] investigating the atomistic origins of the ultralow friction coefficient of a molybdenum disulfide (MoS_2_) coating in ultrahigh vacuum conditions. The study of superlubricity gained momentum in the following years, with a significant focus on nanoscale phenomena, where friction becomes dependent on atomic interactions and quantum effects. This field has since evolved, covering a broader array of materials, and exploring the potential to achieve superlubricity at the macroscale.

Interestingly, superlubricity is not exclusive to any specific class of materials. It has been observed in various systems, ranging from traditional layered materials such as graphite [6,7] to advanced engineering composites [8,9] and diamond-like carbons [10,11]. This versatility points to a future where materials can be engineered for superlubricity to suit specific applications. Delving deeper into the material science of superlubricity unlocks the potential for novel applications and industries that can significantly benefit from near-zero friction.

Achieving superlubricity is a complex endeavor, dependent on multiple factors, including material properties, surface structures, environmental conditions, and even the scale at which it is studied. It can manifest in various forms, such as structural superlubricity [12,13], where the alignment of atoms on the sliding surfaces reduces friction, or thermolubricity [14,15], where temperature changes lead to a near-zero friction state. An understanding of these different mechanisms is crucial for harnessing the full potential of the superlubricity phenomenon.

Superlubricity carries immense potential for application across different domains. Mechanical systems stand to significantly gain from the reduced energy loss and increased operational life. Industries reliant on heavy machinery, such as manufacturing, construction, and transportation, can benefit from the reduced wear and tear, leading to significant cost savings. Furthermore, the energy sector could leverage superlubricity to enhance the efficiency of energy generation and transmission systems. Additionally, the field of biomedicine can benefit from the reduced friction in medical devices and implants, improving their longevity and patient comfort. In recent years, there has been growing interest in the use of low-dimensional materials in biomedicine and implantable devices to achieve superlubricity. These materials, such as graphene and molybdenum disulfide, exhibit unique properties and offer potential advantages for achieving superlubricity in biomedical implants, which can significantly reduce friction and wear, leading to enhanced functionality and durability. However, concerns arise regarding the potential impact of these low-dimensional materials on human biology, specifically in relation to coagulation and thrombosis. Understanding the interactions between these materials and blood components and cells is essential for ensuring the safety and efficacy of implantable devices.

Despite the promising potential, superlubricity is not without its challenges. Technical difficulties in achieving and maintaining the superlubric state, particularly at the macroscale, present significant obstacles. Additionally, scaling up superlubricity for industrial applications poses a considerable challenge. Sustainability concerns, such as the environmental impact of creating superlubric materials and the energy costs involved, further complicate matters. Therefore, this comprehensive review serves as a reference for researchers and industry professionals involved in the advancement and utilization of superlubric materials.

## 2. Background

The study of superlubricity serves as a cornerstone of advanced tribology, focusing on the underlying mechanisms that lead to near-zero friction states. This background section provides an essential context, providing insights into the definition and basic principles of superlubricity, the history and evolution of superlubricity research, and different types of superlubricity. The comprehension of these aspects is pivotal for understanding the progression of superlubricity as a scientific concept and its potential applications in various fields.

### 2.1. Definition and Basic Principles of Superlubricity

**Figure 1:**
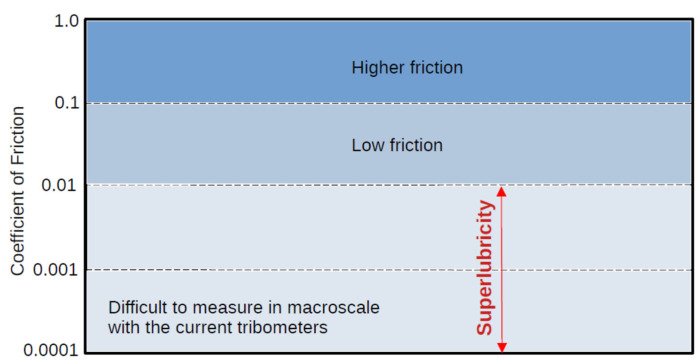
Range of the coefficient of friction and superlubricity.

Superlubricity, colloquially known as ‘zero friction’, is a physical state wherein the friction between two surfaces reaches minimal or near-zero levels. It is a phenomenon that defies our traditional understanding of friction and has significant implications for energy efficiency, wear resistance, and material longevity. Figure 1 shows the state of superlubricity with respect to the range of the friction coefficient. The definition of superlubricity varies depending on the context and application. However, in general, a friction coefficient of less than 0.01 is often considered indicative of superlubric behavior. This extremely low friction coefficient signifies the presence of an effective lubrication mechanism that allows for nearly frictionless sliding between surfaces. It is important to note that specific materials, surface conditions, and operating parameters can influence the friction coefficient and the threshold at which superlubricity is achieved. Therefore, the exact value considered as superlubricity may vary in different experimental setups and systems.

At its core, superlubricity is typically characterized by a structural lattice mismatch at the atomic or molecular level [16]. This incommensurability principle suggests that when two crystalline surfaces with misaligned or mismatched lattice structures slide against each other, they present minimal contact points, thus dramatically reducing friction. For example, Mandelli et al. [17] investigated the sliding friction of heterojunctions formed by graphene and hexagonal boron nitride and demonstrated that when the lattice structures of these two materials were misaligned at a certain angle, the system transitioned to a superlubric state. Graphene is known for its exceptional smoothness at the atomic level, as it consists of a single layer of carbon atoms arranged in a hexagonal lattice. The atomic structure of graphene results in its exceptional lubricity and low friction properties. Graphene’s smooth atomic surface enables it to easily slide against other materials, exhibiting superlubricity. The absence of a lattice mismatch or surface irregularities reduces friction and promotes the efficient sliding of graphene layers.

Superlubricity can also occur through the formation of a third-body layer. This principle involves an intermediary layer of nanoparticles, molecules, or even ionic liquids that separate the two primary surfaces, thus reducing the contact area and friction. For example, Wang et al. [18] used ionic liquids to reduce friction to near-zero levels, even under a high load capacity. This simple yet effective method of achieving liquid superlubricity underscores the potential of ionic liquids in enhancing the efficiency of mechanical systems.

**Figure 2:**
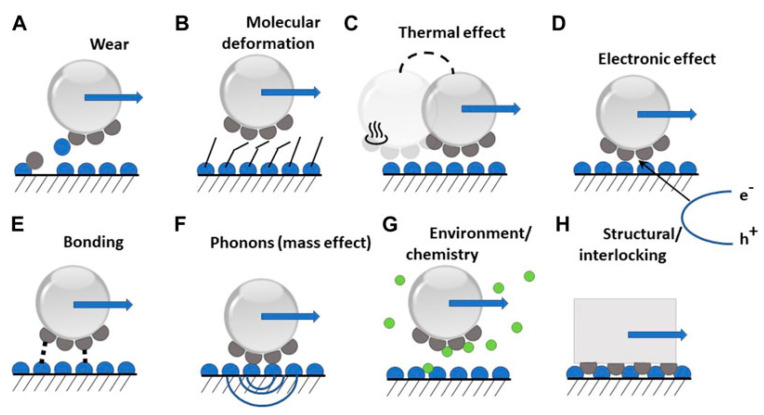
Schematic representations of various mechanisms associated with energy dissipation during sliding, including: (**A**) wear, (**B**) molecular deformation, (**C**) thermal effect, (**D**) electronic effect, (**E**) bonding, (**F**) phonons, (**G**) environment/chemistry, and (**H**) structural/interlocking [20]. Copyright © 2023 American Chemical Society.

Environmental factors also critically influence the realization of superlubricity. Aspects such as temperature, humidity, pressure, and the presence of specific gases or liquids can trigger or enhance superlubric conditions. While these factors play a critical role in superlubricity, it is essential to consider intervals or modes where the properties of superlubricants may deteriorate. Extreme temperatures, high humidity levels, and incompatible pressures or media can diminish the superlubrication’s effectiveness. Thus, precise control and consideration of these environmental parameters are necessary for achieving and maintaining the desired superlubric behavior in practical applications. For example, Kim et al. [19] highlighted how superlubricity in titanium-doped diamond-like carbon on a steel tribopair could be accomplished under specific humidity and temperature settings. To achieve a superlubric sliding regime, numerous solutions focus on mitigating frictional energy dissipation mechanisms. Friction is typically linked to wear, where energy is expended to initiate damage on the sliding surfaces, leading to the formation of cracks and defects [20,21]. Figure 2 shows the schematics of possible mechanisms for energy dissipation during sliding. The pursuit of superlubricity has been a longstanding and highly desirable goal, yet it remains a significant challenge due to the multitude of factors that contribute to friction losses, as illustrated in Figure 2.

In essence, superlubricity is an emergent phenomenon that combines material science, physics, and tribology, requiring a keen understanding of atomic structures, surface interactions, and environmental conditions. While it presents its challenges, the research into superlubricity is not only academically intriguing, but also has the potential to revolutionize numerous fields, from microelectronics to mechanical engineering and beyond.

### 2.2. Evolution of Superlubricity Research

As mentioned in the previous section, the exploration of superlubricity, or the state of near-zero friction, commenced in the early 1990s, with the pioneering work of Hirano and Shinjo [4,22]. They identified the superlubricity phenomenon while investigating the tribological properties of mica surfaces and noticed that under certain conditions, the friction between mica surfaces approached near-zero levels. This important discovery started a new chapter in the study of friction and materials, kicking off the beginning of research into this ultra-low-friction state known as superlubricity.

Following this pioneering work, the initial research primarily focused on layered materials such as graphite, mica, and other lamellar structures. The unique structural attributes of these materials, especially their incommensurate lattice structures, were identified as key facilitators of superlubricity [20].

The subsequent decade saw the exploration of superlubricity in a broader array of materials. Scientists extended their research to diamond-like carbons (DLCs) and other synthetic materials, thanks to Erdemir’s research in the 2000s [23,24] that showed DLCs could achieve superlubricity under certain environmental conditions. Around this time, research started to broach the possibility of achieving superlubricity at the macroscale. This marked a significant shift in the field, from being a nanoscale phenomenon to a potentially game-changing concept with wide-scale applications.

The advent of advanced nanotechnology and material science tools has transformed our understanding of superlubricity. Advanced microscopy techniques, such as scanning tunneling microscopy (STM) and atomic force microscopy (AFM), have made it possible to observe and manipulate atomic interactions, offering new insights into the mechanics of superlubricity. For instance, Liu et al. [25] proposed a method using heat-aided physical separation and transfer to create different 2D flake-covered tools for AFM. This way, we can directly measure the friction between 2D flakes that are in contact at the single-crystal level.

Now, the exploration of superlubricity extends well-beyond the boundaries of physics and material science. It is a multidisciplinary field, encompassing chemistry, engineering, and biomedicine. Researchers are investigating superlubricity’s potential in areas such as drug delivery, where nanoparticles exhibiting superlubricity could offer minimal tissue damage [26], and energy production, where superlubricity could enhance the efficiency of mechanical systems [27].

Superlubricity research has come a long way since its inception in the 1990s. With each passing year, we uncover new facets of this phenomenon, bringing us a step closer to realizing its potential in transforming a wide range of scientific and technological domains.

### 2.3. Different Types of Superlubricity

The state of superlubricity can materialize in several forms, each associated with distinctive conditions and underlying mechanisms. By understanding these distinct types, researchers can effectively tailor conditions to achieve superlubricity in a variety of systems. Here, we detail four recognized types of superlubricity: structural, thermolubricity, quantum, and ionic.

*Structural Superlubricity:* This form of superlubricity stems from the incommensurate contact between atomically smooth surfaces. When the atomic structures of the sliding surfaces lack matching periodicity, it reduces their interactive forces, leading to ultra-low friction. Graphene, with its unique lattice structure, is an archetypal example of a material demonstrating structural superlubricity when its layers are misaligned [13,28]. Interestingly, misalignment between the layers of graphene can result in the formation of twisted graphene structures, commonly known as twisted bilayer graphene (TBG) or moiré superlattices. When two graphene layers are stacked with a slight rotational mismatch, a moiré pattern emerges due to the interference of the overlapping carbon atoms [29]. This twisting creates a new periodicity, altering the electronic and mechanical properties of graphene, including its lubrication behavior. Furthermore, by reversing the layers at specific angles, multilayer graphene with a reversed stacking order can be achieved, introducing additional structural complexities that can influence the superlubricity characteristics. Understanding the interplay between the misalignment, moiré patterns, and the resulting superlubric behavior of graphene structures holds significant potential for advancing our knowledge of structural superlubricity and the design of novel lubricating materials.

**Figure 3:**
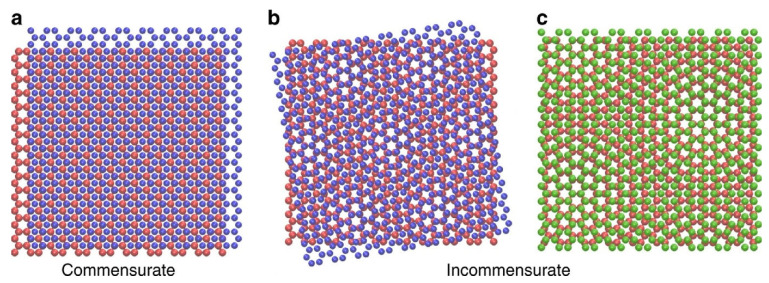
Schematic representation of structural superlubricity. (**a**) Commensurability, where matching rotational symmetry and orientation prevent sliding. (**b**) Incommensurability (angular rotation): disrupts the symmetry, enabling sliding. (**c**) Incommensurability (lattice spacing mismatch): the layers have a mismatch in the lattice spacing, allowing for sliding due to the absence of interlocking [29]. Copyright © 2023 Springer Nature.

Figure 3 illustrates the concept of structural superlubricity. In Figure 3a, when two layers possess identical rotational symmetry and orientation, their lattices interlock, preventing sliding (referred to as commensurability). In Figure 3b, one layer is subjected to an angular rotation, disrupting the rotational symmetry. In Figure 3c, the layers exhibit a lattice spacing mismatch, leading to incommensurability. Both Figure 3b,c represent scenarios where sliding is possible due to the absence of interlocking between the layers [29].

*Thermolubricity:* This type of superlubricity pertains to the reduction of friction due to changes in temperature. When materials or lubricants are subjected to specific temperature ranges, their properties can undergo alterations, leading to a superlubric state. For example, Bai et al. [14] studied the friction pattern of graphene with various layers at temperatures ranging from 100 to 300 K and investigated thermolubricity in cold environments using graphene. At the heart of thermolubricity is the concept of temperature-dependent material properties. As temperature changes, the physical and chemical interactions within the material alter, affecting its lubrication behavior. These temperature-induced modifications can involve variations in surface roughness, adsorbed species, surface diffusion, and interfacial interactions.

In the case of graphene, temperature can impact its interlayer interactions and the mobility of adsorbates on its surface. The study by Bai et al. sought to elucidate how these temperature-driven changes affect the frictional behavior of graphene and its potential for achieving superlubricity in cold environments. Understanding thermolubricity is vital for optimizing lubricated systems across various technological applications. By exploiting temperature as a parameter to modulate friction and wear, it becomes possible to design tailored lubricants and solid-state interfaces that exhibit enhanced performance under specific temperature conditions. Furthermore, the exploration of thermolubricity sheds light on the fundamental principles underlying friction and lubrication, contributing to the advancement of tribology and the development of novel lubrication strategies.

*Quantum Superlubricity:* Predominantly observed at the nanoscale, where quantum mechanical effects become substantial, this form of superlubricity exploits the wave-like nature of particles at this scale. It results in non-contact friction, where surfaces ‘slide’ over each other without actual physical contact [20]. This type of superlubricity is still in its theoretical and experimental infancy, but it holds intriguing potential.

At the nanoscale, quantum mechanical effects such as wave interference and quantum tunneling come into play, allowing surfaces to effectively “slide” over each other without physical contact. By exploiting delicate quantum interplays, such as the suppression of phonon-mediated interactions or the preservation of coherent wave-like states, non-contact friction can be achieved. However, practical implementation and widespread application of quantum superlubricity face significant challenges. The precise control and manipulation of quantum states at the nanoscale, along with the mitigation of external factors that can disrupt quantum coherence, are complex tasks that need to be addressed. Nonetheless, the exploration of quantum superlubricity opens up exciting possibilities for frictionless nanoscale technologies. Understanding the underlying mechanisms and developing strategies to harness and optimize this quantum effect could lead to the design of novel lubrication strategies and the creation of frictionless nanoscale devices.

*Ionic Superlubricity:* This category of superlubricity is characteristic of ionic liquids, where the presence of ions helps reduce friction. Ionic liquids are unique compounds composed of large organic cations and small inorganic or organic anions, and they exhibit remarkable lubricating properties. In a notable study, Chen et al. [30] developed a new series of lubricants, known as polymer-based ionic liquids, that have a flexible and electron-rich polymer chain connected to the cation. They showed that these lubricants provide much lower friction and better protection for surfaces compared to the traditionally used lubricants. Ionic superlubricity is particularly significant in biological systems, where ionic liquids naturally exist and contribute to their low frictional properties. The presence of ions in biological fluids and tissues facilitates the formation of lubricating layers that enable smooth movement and minimize wear between interacting surfaces. Understanding and harnessing the lubricating capabilities of ionic liquids in biological systems can have profound implications for the development of biocompatible lubricants and biomimetic lubrication strategies.

Each form of superlubricity involves unique conditions and mechanisms, offering possibilities to achieve superlubricity across a broad spectrum of systems and scales. The unifying feature of these categories is the ultra-low friction, which opens the door to different applications across technology and industry, enhancing the energy efficiency and device performance.

Importantly, these categories should not be seen as isolated, as there is considerable overlap and interplay among them. For instance, structural superlubricity at the nanoscale may invoke quantum effects, and thermolubricity could induce changes in the structural properties of materials. This interconnectedness underscores the complexity of superlubricity and emphasizes the need for a holistic understanding of the various forms and governing principles of this phenomenon.

## 3. Mechanics of Superlubricity

The mechanics of superlubricity involves the interplay of complex physical and chemical phenomena at different scales, from atomic/molecular structures to macroscopic interactions. The complex dynamics of superlubricity are dictated by various factors, including material properties, structural alignment, environmental conditions, and the scale of observation. In this section, we delve into the role of atomic/molecular structures in superlubricity, explore the concept at nano- and macro-scales, and discuss the mathematical models that have been developed to describe and predict superlubricity.

### 3.1. The Role of Atomic/Molecular Structures in Superlubricity

Atomic and molecular structures have a pivotal role in dictating superlubricity in various materials. The intricate spatial arrangements of atoms or molecules in a material can either enable or inhibit superlubricity, often governed by the principle of lattice mismatch [31,32].

In the case of graphite, which was one of the first materials where superlubricity was observed, the atomic structure plays a key role. Graphite consists of graphene layers, wherein each layer consists of carbon atoms arranged in a hexagonal lattice. Despite their identical lattice structures, the relative arrangement of these layers can be shifted, rendering their interaction incommensurate, which is a primary condition for superlubricity. Buzio et al. [33] showed that the friction between two layers of graphite could be nearly eliminated when the surfaces were slid at a specific angle, highlighting the importance of the atomic structure and its alignment.

Similarly, DLC has been extensively studied for its superlubricity properties. DLC’s structure contains a mixture of sp^2^ (graphitic) and sp^3^ (diamond-like) hybridized carbon, contributing to its unique mechanical properties [23]. A study by Yu et al. [34] showed that a multilayer film with MoS_2_ and DLC layers on hydrogenated DLC surfaces could achieve superlubricity in damp air.

The atomic structure’s significance extends beyond solid materials. In the context of lubricants, certain structures can reduce friction by forming an intermediary layer, often referred to as a ‘third-body layer’. The formation of this intermediary layer is largely influenced by the molecular structure of the lubricant. A study by Gao et al. [35] demonstrated that certain lubricants, due to their unique molecular structures, could align themselves to form highly ordered ‘liquid crystals’ at the interface, effectively reducing the contact area between the surfaces, and thus enabling superlubricity.

Additionally, the formation of a tribofilm, a thin film resulting from mechanical interaction, can contribute to superlubricity. A study by Yi et al. [36] demonstrated that by introducing Ti_3_C_2_T_x_ MXene nanoflakes in glycerol at Si_3_N_4_/sapphire interfaces, a tribofilm will be formed that separates the two surfaces, drastically reducing friction.

Further research has highlighted the possibility of superlubricity in biological systems. For example, the unique molecular structure of synovial fluid—the ‘lubricant’ in human joints—could form a lubricating layer that minimizes friction, suggesting a form of superlubricity [37].

In essence, the atomic and molecular structures in various materials, from graphite and DLC to lubricants and biological fluids, play an essential role in achieving superlubricity. Recent advancements in research have provided a deeper understanding of these structures, and how they interact under various conditions. This understanding paves the way for the design and development of new materials and systems that can exploit the phenomenon of superlubricity.

### 3.2. Nanoscale Superlubricity

Nanoscale superlubricity, the exploration of near-zero friction at the atomic level, is a fast-expanding domain, with recent research uncovering new insights into the atomic interactions and quantum effects that can promote superlubricity.

One of the most compelling examples of nanoscale superlubricity is exhibited by carbon nanotubes (CNTs). These cylindrical structures composed of carbon atoms have demonstrated exceptional superlubric properties when a smaller tube is inserted into a larger one, creating a ‘telescoping’ effect. Li et al. [38] demonstrated this phenomenon, with double-walled CNTs exhibiting superlubricity due to incommensurate contact and a minimal number of interacting atoms, leading to ultra-low friction.

Graphene, a one-atom-thick layer of carbon atoms arranged in a hexagonal lattice, has also been a prominent material in nanoscale superlubricity research. Recent studies have shown that by manipulating the alignment of graphene layers, superlubricity can be achieved. Cheng et al. [39] demonstrated that two graphene layers in sliding commensurate contact, which are subjected to an isotropic, in-plane, synchronous strain, induce superlubric behavior.

In addition to solid materials, investigations into nanoscale superlubricity have extended to liquids, particularly ionic liquids. These compounds consist of positively and negatively charged ions and can form highly ordered structures at the interface with a surface, serving as an effective ‘liquid ball bearing’. A study by Zheng et al. [40] explored the superlubric capabilities of ionic liquids, demonstrating that the ions could self-assemble into layers at the interface with a surface and significantly reduce friction.

Moreover, advancements in nanotechnology have enabled researchers to explore superlubricity in nanostructured materials. A study by Deng et al. [41], for instance, demonstrated superlubricity in a set of nanostructured multilayer Si-DLC/PLC films in high-load working environments. With these nanostructured films, the researchers were able to manipulate the material to achieve structural superlubricity at the nanoscale.

Nanoscale superlubricity offers a unique avenue to achieve ultra-low friction, exploiting atomic-scale phenomena that may not be significant or observable at larger scales. With recent advancements in nanotechnology and materials science, our understanding of nanoscale superlubricity has grown, leading to promising opportunities in various fields, including energy, computing, and nanotechnology. The future of nanoscale superlubricity holds great potential and may well revolutionize how we understand and apply the principles of friction and lubrication.

### 3.3. Macroscale Superlubricity

Macroscale superlubricity, the pursuit of achieving near-zero friction at larger scales, is a rapidly developing area in superlubricity research. The transition from nano- to macro-scale has been fraught with complexities, mainly due to the increase in the number of interacting atoms and the environmental variables that come into play. However, successful examples in recent years have shown that it is a feasible pursuit, with substantial implications for a wide range of applications, from energy conservation to industrial and mechanical systems.

One of the key advances in macroscale superlubricity research is the utilization of DLC coatings with hydrogen termination. The carbon atoms in DLC undergo sp^2^ and sp^3^ hybridizations, resulting in a complex, non-uniform landscape that can induce superlubricity. For example, Liu et al. [42] investigated the superlubricity stability of hydrogenated DLC film in a vacuum environment and demonstrated that a relatively stable macroscale superlubricity state can be maintained at low sliding velocities. This research showed the potential for DLC coatings in applications such as automotive components and electronic devices.

In addition to the material’s characteristics, the environment can play a crucial role in macroscale superlubricity. Yin et al. [43] investigated the macroscale superlubricity of multilayered MoS_2_–Ag film in a cryogenic environment against a bare steel ball under a high load. The results demonstrated that the MoS_2_–Ag film could withstand pressure exceeding 2 GPa, preserving an exceptionally low friction coefficient of less than 0.001 at 170 Kelvin. The role of specific gases has also been investigated. A study by Wang et al. [44] demonstrated macroscale superlubricity in amorphous, hydrogenated carbon films under an atmosphere of nitrogen. The research suggested that nitrogen molecules adsorbed on the contact interface and acted as tiny ball bearings, facilitating the sliding motion.

Moreover, the role of third-body materials in macroscale superlubricity has been explored. For example, it has been shown that with molybdenum disulfide as a lubricant, superlubricity could be achieved in a steel-on-steel system at the macroscale, as MoS_2_ forms a tribofilm on the contact surfaces, effectively separating them and reducing friction [8,43].

In summary, the pursuit of macroscale superlubricity, while challenging, has been gathering momentum. The blend of advancements in material science, environment manipulation, and the innovative use of third-body materials holds promise for the attainment of this phenomenon at larger scales. This progress in understanding macroscale superlubricity is not just academically exciting but also offers significant potential for industrial applications, energy efficiency, and sustainability.

### 3.4. Mathematical Models for Superlubricity

Mathematical modeling has been instrumental in exploring and comprehending the underlying principles of superlubricity. By providing a theoretical framework, mathematical models have paved the way to predict conditions favoring superlubricity, as well as contributing to experimental research.

The Prandtl–Tomlinson (PT) model, one of the most well-known friction models, has been adapted to include superlubricity [39,45]. The PT model was initially designed to explain friction at the atomic level, based on the concept of a particle moving on a periodic potential. To accommodate superlubricity, the model has been extended to consider structural lattice mismatch and the impact of external factors such as temperature and load. Research by Buzio et al. [45] applied the single-asperity PT model for investigating a load-driven, atomic-scale transition from dissipative stick–slip motion to smooth sliding (indicative of superlubricity) in mesoscopic graphite contacts.

Another widely used model is the Frenkel–Kontorova (FK) model [46,47], which is particularly apt for examining systems with competing length scales, such as the mismatch of atomic lattices seen in structural superlubricity. The FK model is capable of capturing the dynamic behaviors of these systems, making it possible to predict conditions that may lead to superlubricity. Han et al. [46] used the FK model to study the interacting atoms arranged on a two-dimensional hexagonal lattice. They investigated the effects of the direction and the magnitude of the external driving force, the adhesive force from the substrate, the interaction strength between atoms in the upper layer, and the misfit angle between two layers on superlubricity.

Yet another model worth mentioning is the van der Waals-corrected density functional theory (DFT). For instance, Sun et al. [16] used the DFT model to predict superlubricity in layered materials such as graphene. This model takes into account the weak van der Waals forces between layers and has been successful in describing the onset of superlubricity in these systems.

Although these mathematical models provide valuable insights into superlubricity, they often simplify the physical and chemical interactions involved in the phenomenon. Consequently, these models might not entirely capture the intricacies of superlubricity. Despite this, the development of mathematical models remains vital to understanding the basic mechanisms underpinning superlubricity. These models not only offer theoretical guidance to experimentalists, but also aid in the design and development of materials and systems capable of superlubric behavior. Hence, the role of mathematical modeling in the progress of superlubricity research continues to be indispensable and is likely to evolve alongside experimental advancements.

## 4. Materials Science in Superlubricity

Material science plays an integral role in the study of superlubricity, providing insights into the conditions and characteristics required for a material to exhibit this phenomenon. The unique attributes of certain materials, coupled with advancements in nanotechnology, have allowed researchers to manipulate and exploit superlubricity for a wide range of applications, from minimizing energy consumption in mechanical systems to reducing wear and tear in industrial components.

The understanding of superlubricity has significantly evolved over the years, thanks to extensive research focusing on the role of material characteristics, atomic structures, and environmental conditions. Researchers have been able to demonstrate superlubricity in a variety of materials, including naturally occurring ones such as graphite and molybdenum disulfide (MoS_2_), and synthetic ones such as diamond-like carbon (DLC). Moreover, the advent of two-dimensional (2D) materials and the ability to engineer material surfaces at the atomic level have opened new avenues for superlubricity research.

In addition, the development of advanced synthetic materials and composites has allowed researchers to design and tailor material properties to enhance their potential for superlubricity. By optimizing these materials at the atomic level, scientists can control friction and wear characteristics, thereby enhancing the performance and efficiency in numerous applications.

The exploration of material science in superlubricity promises to spur advancements not only in fundamental science, but also in various technological and industrial sectors. Understanding and exploiting the principles of superlubricity could revolutionize numerous fields, including manufacturing, transportation, and energy generation, by significantly reducing energy consumption and extending the lifespan of components.

### 4.1. Characteristics of Materials Exhibiting Superlubricity

Materials capable of exhibiting superlubricity possess certain distinctive characteristics that set them apart. These include the atomic structure, surface smoothness, environmental adaptability, and specific mechanical properties. As these play a critical role in the phenomenon of superlubricity, recent research and advancements have broadened the understanding of these characteristics, pushing the boundaries of how materials are engineered for low-friction applications.

*Atomic Structure:* The atomic structure of a material significantly influences its ability to exhibit superlubricity. For instance, Zheng et al. [48] explored the superlubricity potential of hexagonal boron nitride (h-BN), a material with a hexagonal atomic lattice structure similar to graphite. The researchers found that when layers of h-BN were rotated relative to each other, the atomic mismatch resulted in superlubricity due to a lack of locking between the layers.

*Surface Smoothness:* The smoothness of a material’s surface at the atomic level is a crucial characteristic that can promote superlubricity. For example, Song et al. [49] used an ultrahigh-vacuum atomic force microscope to carefully control and study the friction between a single layer of MoS_2_ and a smooth gold (111) surface. The atomically smooth surface of gold helped establish near-zero friction conditions, providing a critical insight into the mechanisms of superlubricity.

*Environmental Adaptability:* How a material responds to changes in its environment is key to its potential for superlubricity. A study conducted by Kim et al. [19] demonstrated that the friction between Ti-doped DLC and steel surfaces could transition to superlubricity under specific humidity and temperature conditions. The researchers discovered that the increased presence of water molecules in a humid environment could form a lubricating layer between the surfaces, significantly reducing friction.

*Mechanical Properties:* The hardness, elasticity, and shear strength of a material can dramatically impact its superlubricity potential. Previous studies discovered that certain soft polymeric materials could exhibit superlubricity when combined with specific lubricants. Despite the low shear strength of the polymer, the presence of the lubricant allowed the surfaces to slide past each other with near-zero friction, demonstrating how adjusting the mechanical properties of a material can enable superlubricity. For instance, Cai et al. [50] showed that superlibricity can be achieved between poly(vinylphosphonic acid)-modified Ti-6Al-4V and polystyrene, lubricated with monovalent salt solutions.

Understanding these characteristics and their interplay is key to identifying potential superlubric materials and designing new ones. By leveraging the principles of materials science, researchers can engineer materials at the atomic level to enhance their superlubricity potential.

### 4.2. Prominent Superlubric Materials

Superlubric materials represent an exciting frontier in the field of materials science, displaying an ability to achieve a nearly frictionless interaction between surfaces. This characteristic opens a vast array of potential applications, spanning from nanotechnology to large-scale industrial machinery, with a promise of a significantly enhanced efficiency and lifespan. Superlubric materials, distinguished by unique atomic structures and specific mechanical and environmental properties, create conditions under which interlocking between surfaces during sliding is drastically reduced or virtually eliminated. The research and development of these materials is a growing field, providing promising avenues for overcoming challenges associated with friction, wear, and energy efficiency in numerous technological applications. As the understanding of superlubricity deepens, researchers continue to explore a variety of materials, both naturally occurring and synthetically created, that exhibit this rare trait. Table 1 lists some materials exhibiting superlubric behavior, including their atomic structure, surface smoothness, and environmental adaptability. Key among these are graphite, diamond-like carbon (DLC), and certain two-dimensional (2D) materials, such as molybdenum disulfide (MoS_2_). The continued exploration and development of superlubric materials promise to drive innovation and improvement across a wide range of industries.

*Graphite:* Recognized as the first material where superlubricity was observed, graphite’s potential continues to be explored in recent research. Its layered atomic structure, in which adjacent layers lack repeated patterns, promotes reduced friction during sliding motions. By using a mix of atomic force microscopy and density functional theory, Shi et al. [51] studied the superlubricity of a graphite surface when it rubs with different types of AFM probes. They showed that an increase in friction happens because of stronger adhesive interactions, which occur due to a higher density of charges at the interface.

*Diamond-Like Carbon (DLC):* DLC, with its unique carbon atomic arrangement, is another prominent superlubric material. The complex configuration provides an uneven atomic landscape that enables structural superlubricity under certain conditions. Liu and Zhang [11] examined the properties and mechanics of superlubricity in DLC film in a vacuum, under both rotary and reciprocating motions. Their findings suggest that film exhibits a steadier state of superlubricity and less wear in rotary motion, which is due to the removal of tribofilm under reciprocating motion.

*MoS_2_ and Other 2D Materials:* Molybdenum disulfide (MoS_2_), with its weak interlayer bonding, is another well-known superlubric material. The ease of sliding between its layers leads to extremely low friction, making it a promising candidate for superlubric applications. Previous studies demonstrated that by precisely controlling the relative alignment of MoS_2_ layers, superlubricity could be achieved and controlled [52]. Other 2D materials, such as hexagonal boron nitride (h-BN) and specific transition metal dichalcogenides (TMDs), have also shown superlubric potential. For example, Song et al. [53] demonstrated superlubricity in h-BN due to its unique incommensurate lattice structure, similar to the phenomenon observed in graphite. These studies show that 2D materials, with their unique characteristics, offer a rich field for further superlubricity research and potential practical applications.

### 4.3. Advanced Synthetic Materials and Composites

The domain of advanced synthetic materials and composites has proven to be a fertile ground for exploring and achieving superlubricity. The ability to manipulate the atomic and molecular structure of these materials allows researchers unprecedented control over their frictional properties, thus enabling innovative applications across diverse fields. One example comes from the work of Zhang et al. [9], where they synthesized amorphous carbon nanocomposites incorporating tantalum carbide nanoparticles. They found that this unique combination of materials achieved superlubricity with the aid of atomic-level dispersed gold atoms under ambient conditions, a crucial requirement for many practical applications. The synergy of the carbon materials’ low shear strength and the gold’s electronic properties resulted in dramatically reduced friction in the macroscale.

Polymer composites represent another promising category of superlubric materials. For example, polytetrafluoroethylene (PTFE) composite doped with nanoparticle additives exhibited exceptional low friction and high wear resistance, making it suitable for flexible electronics and wearable devices that require both durability and flexibility [40,54].

An especially exciting development in the field is the emergence of self-lubricating materials. For example, self-lubricating composites integrating molybdenum disulfide (MoS_2_) nanoparticles into a polymeric matrix maintain ultra-low friction even under severe operating conditions, eliminating the need for external lubricants [55,56]. This self-lubricating capability is particularly beneficial in environments where applying or replenishing lubricants is challenging or impossible, such as in space applications.

The development of advanced synthetic materials and composites for superlubricity is a rapidly expanding area of research, presenting numerous promising avenues for technological advancements. As our comprehension of superlubricity’s underlying mechanisms deepens, we can anticipate an increasing pace of innovations in material design, leading to a broader spectrum of applications and improvements in energy efficiency and equipment longevity.

## 5. Techniques for Achieving Superlubricity

Superlubricity is a complex physical state that depends on an intricate balance of conditions. Researchers have developed various techniques for achieving this desirable low-friction condition, often manipulating the material properties, scale, and environmental factors. By understanding these strategies and their interplay, we can push the boundaries of our technological capabilities, improve the efficiency and lifespan of mechanical systems, and reduce the energy consumption and environmental impact of numerous industries.

The techniques for achieving superlubricity broadly fall into three categories: nanoscale techniques, macroscale techniques, and the influence of environmental conditions. Each category has a unique set of challenges and opportunities and has been a focal point of intense research and development.

At the nanoscale, the manipulation of atomic and molecular structures is key to achieving superlubricity. Through careful design and control of material interfaces at the atomic level, scientists have been able to observe and harness superlubricity under specific conditions. However, translating these nanoscale successes to the macroscale has proven to be a formidable challenge.

At the macroscale, researchers strive to replicate the ultra-low-friction states observed at the nanoscale in larger, real-world applications. This involves not only the scaling of material structures and interactions, but also the consideration of operational and environmental conditions that could affect superlubricity.

Environmental conditions play a critical role in enabling superlubricity. Factors such as temperature, humidity, and pressure can profoundly influence the frictional properties of a system. Understanding and controlling these factors can make the difference between normal friction and superlubricity.

While many advances have been made in developing techniques for achieving superlubricity, the field is still relatively young, and there is much more to learn. As our understanding of the phenomenon deepens and our experimental and computational tools become more sophisticated, we can expect to see exciting new developments in this promising area of research.

### 5.1. Nanoscale Techniques

At the nanoscale, where quantum effects and atomic interactions dominate, researchers have developed techniques that leverage the unique properties of materials to achieve superlubricity. These techniques involve careful manipulation of atomic structures, interfaces, and chemical environments to reduce friction at the atomic level.

One effective technique for achieving superlubricity at the nanoscale is through the layering and rotation of two-dimensional (2D) materials. Graphene, a single layer of carbon atoms arranged in a hexagonal lattice, has been a focal point of research in this area. For example, by rotating layers of graphene relative to each other, a state of structural superlubricity could be achieved [57]. The misalignment of the graphene layers disrupts the lattice matching, preventing interlocking and leading to ultra-low friction. This technique, known as twistronics [58], provides a means to precisely control friction by manipulating the orientation and relative alignment of layered materials. By changing the twist angle between graphene layers, it is possible to modulate the electronic properties of the system. The rotational alignment of the layers leads to the formation of moiré patterns [52,58], which alter the electronic band structure of the unfolded graphene system.

Another nanoscale technique involves the use of nanoparticles as a third-body layer to separate contacting surfaces. For example, a study conducted by Wang et al. [59] applied hydroxide nanoparticles and nanosheets as lubricating additives in polyalkylene glycol aqueous solutions for steel. The presence of the nanoparticles created a nanoscale separation, reducing the real contact area, and thus minimizing friction. This approach exploits the unique properties of nanoparticles to act as lubricating agents and enhance superlubricity at the atomic scale.

Controlling the chemical environment is another effective nanoscale technique for achieving superlubricity. By applying a thin layer of a specific ionic liquid to a surface, researchers can create a lubricating layer that facilitates low-friction sliding. For example, Zhang et al. [60] demonstrated the achievement of ionic superlubricity by applying ionic liquids to a graphite surface. The ionic liquid formed a lubricating layer that reduced interfacial adhesion and enabled the graphite layers to slide over each other with minimal resistance. This technique highlights the role of tailored chemical environments in manipulating atomic-scale interactions and reducing friction.

These nanoscale techniques exemplify the potential of atomic-scale manipulations in achieving superlubricity. By carefully controlling atomic structures, interfaces, and chemical environments, researchers can minimize interfacial adhesion and enable near-frictionless sliding. However, the challenge lies in scaling up these nanoscale techniques to achieve superlubricity in larger, macroscale systems, where additional factors such as surface roughness, load, and environmental conditions come into play. Overcoming these challenges is a crucial area of ongoing research, with the potential to revolutionize various technological applications.

### 5.2. Macroscale Techniques

**Figure 4:**
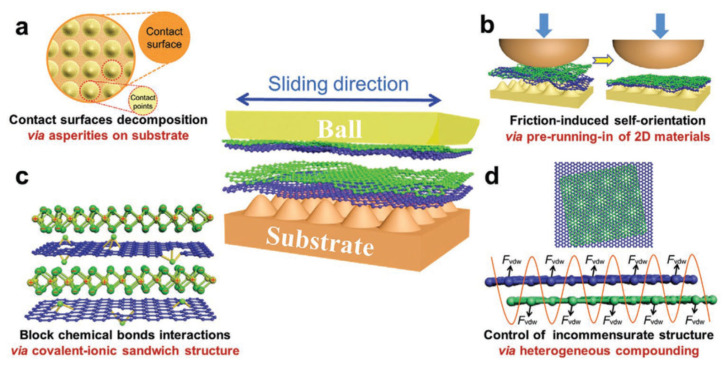
Mechanism of macroscale superlubricity. (**a**) Macroscale contact divided into microscale point contacts. (**b**) Formation of a layered structure. (**c**) Heterogeneous nanocomposite blocking chemical interactions. (**d**) Incommensurate contact, where van der Waals force (Fvdw) allows for low friction [61]. Copyright © 2023 WILEY-VCH Verlag GmbH & Co. KGaA, Weinheim.

Achieving superlubricity at the macroscale presents unique challenges due to the complexity of maintaining atomically smooth surfaces and controlling friction on a larger scale. However, researchers have made significant progress in developing techniques that aim to replicate the low-friction conditions observed at the nanoscale in macroscale systems. These macroscale techniques involve various approaches, such as coatings and surface modifications, the use of lubricants and additives, surface texturing, and the development of composite materials. Figure 4 illustrates various mechanisms involved in achieving macroscale superlubricity. The contact at the macroscale is divided into microscale point contacts, allowing for reduced friction. The formation of a layered structure further contributes to low-friction behavior. Chemical interactions are effectively blocked by the presence of a heterogeneous nanocomposite, preventing energy dissipation. Additionally, incommensurate contact, facilitated by the van der Waals force, enables superlubric behavior [61]. The formation of a tribofilm has a significant impact on superlubricity. When a tribofilm forms at the interface of two sliding surfaces, it acts as a lubricating layer that separates and protects the contacting surfaces and reduces direct contact, preventing surface adhesion and minimizing frictional forces. It also provides a smooth and low-friction surface, contributing to superlubric behavior. The composition and properties of the tribofilm influence its effectiveness as a lubricant, with certain compounds and additives promoting superior superlubricity. Understanding the formation and behavior of tribofilms is essential for optimizing lubrication strategies and designing materials and coatings that facilitate and sustain superlubric conditions.

One prominent approach to achieving macroscale superlubricity is the application of coatings and surface modifications. Diamond-like carbon (DLC) coatings have shown great potential for reducing friction in macroscale systems. DLC coatings, typically deposited using techniques such as physical vapor deposition or plasma-enhanced chemical vapor deposition, provide a smooth and wear-resistant surface that reduces friction between contacting surfaces. Recent research has focused on optimizing DLC coatings by introducing specific elements or incorporating nanostructures to further enhance their superlubric properties. For example, a study conducted by Yin et al. [62] showed that using graphene quantum dot-modified DLC films exhibited enhanced superlubric behavior by reducing surface adhesion.

Another technique involves the use of lubricants and additives to reduce friction in macroscale systems. Table 2 provides a comparison of selected solid and liquid lubricants used for achieving superlubricity, including their friction coefficients, operating temperature ranges, and environmental impact. Researchers have explored various lubricants, including ionic liquids, liquid metals, and self-assembled monolayers, to achieve superlubricity. For instance, Du et al. [63] developed a polyethylene glycol-tannic acid liquid lubricant for a silicon nitride/glass sliding pair. The liquid formed a thin lubricating layer that significantly reduced friction and wear. Similarly, the use of liquid metals, such as gallium-based alloys, has shown promise in achieving superlubricity in macroscale systems by forming a low-shear layer between the contacting surfaces.

Surface texturing is another promising macroscale technique for achieving superlubricity. By designing specific patterns or textures on the surface, researchers can alter the contact area and lubricant trapping, thereby reducing friction. Laser surface texturing and nanoscale patterning are among the techniques employed to create customized surface structures that enable superlubricity. These techniques have demonstrated their efficacy in improving the tribological properties of various materials, including metals, polymers, and ceramics. For example, Vlǎdescu et al. [64] utilized laser surface texturing on polymeric surfaces, resulting in reduced friction and improved wear resistance.

Additionally, researchers have investigated the use of composite materials to achieve macroscale superlubricity. By combining materials with distinct mechanical properties, such as hard nanoparticles embedded in a soft matrix, researchers have been able to create composite systems with reduced friction. For instance, Zhuang et al. [65] developed an epoxy resin coating strengthened with polydimethylsiloxane nanoparticles and could achieve steady superlubricity during the sliding wear tests of this composite coating against a silicon nitride ball. The presence of the nanoparticles reduced the contact area and altered the stress distribution, resulting in superlubric behavior.

These macroscale techniques demonstrate the ongoing efforts to replicate the low-friction conditions observed at the nanoscale in practical, real-world applications. While challenges remain in scaling down the effects observed at the atomic level, advancements in coatings, lubricants, surface texturing, and composite materials provide promising avenues for achieving superlubricity on a larger scale. Continued research and development in these areas will contribute to the advancement of superlubricity and its application in various industries, such as automotive, aerospace, and biomedical engineering.

### 5.3. Influence of Environmental Conditions

Environmental conditions, including temperature, humidity, and surrounding gases or liquids, can have a profound impact on the frictional behavior of materials. Understanding and controlling these environmental factors is crucial for achieving and maintaining superlubricity.

Temperature plays a critical role in superlubricity, as it affects the material’s mechanical properties and the nature of the interfacial interactions. Recent research has investigated the temperature dependence of superlubricity in various systems. For example, Wang et al. [66] examined the temperature effects on the superlubric behavior of an MoS_2_-based coating with magnesium silicate hydroxide and antimony trioxide. Tribological tests conducted in an open-air environment showed that as the testing temperature increased above 200 °C, the friction coefficient of the composite coating sharply decreased, ultimately achieving a state of superlubricity. This was attributed to the synergistic lubrication characteristics of these materials, enabling easy shearing of the film at high-temperature conditions. This research provides insights into the temperature-dependent nature of superlubricity and informs the design of materials for specific operating conditions.

Humidity is another environmental factor that can significantly influence superlubricity. Moisture molecules can act as lubricants or contribute to the formation of lubricating films, reducing friction between surfaces. For instance, Hua et al. [67] achieved superlubricity between steel surfaces lubricated by a mix of ionic liquids and glycerol water solution. They found that the humidity level could act as a control switch between superlubricity and non-superlubricity, and their study introduces a new approach to achieving switchable superlubricity under low-humidity conditions. Understanding the interplay between humidity and material properties is crucial for optimizing superlubric systems in humid environments.

The presence of specific gases or liquids can also impact superlubricity. For instance, Saravanan et al. [68] studied the performance of multilayer polyethylenimine/graphene oxide thin films as solid lubricants in different environments. The researchers found that introducing certain gases, such as nitrogen or hydrogen, between the contact surfaces reduced the friction coefficient, indicating the potential for achieving superlubricity through controlled gas environments. Similarly, studies have explored the influence of various lubricating liquids, such as oils or ionic liquids, on superlubric behavior, highlighting the importance of tailored lubricant selection for achieving low-friction conditions [63,69,70].

In recent years, advancements in environmental control techniques have allowed researchers to explore superlubricity under extreme conditions. For example, experiments conducted in high-vacuum environments or under controlled gas atmospheres [49,68,71] have provided insights into the fundamental mechanisms of superlubricity and its dependence on environmental factors. These studies have contributed to our understanding of how to achieve and maintain superlubricity in specific operating conditions.

Overall, the influence of environmental conditions on superlubricity is a crucial aspect that researchers must consider. By understanding how temperature, humidity, gases, and liquids affect the frictional behavior of materials, scientists can develop strategies to optimize superlubric systems for specific applications and operating environments. This knowledge enables the design of materials and lubricants that can sustain superlubricity under desired conditions, contributing to the improved efficiency, reduced wear, and extended lifespan of various mechanical systems.

## 6. Applications of Superlubricity

Superlubricity, with its ability to significantly reduce friction and wear, has garnered significant interest across various fields. The unique properties and potential of superlubric materials and systems have led to a wide range of applications, from mechanical systems to energy efficiency, biomedical applications, and emerging technologies. Table 3 summarizes selected applications of superlubricity in different industries, along with the specific benefits and advancements achieved in each application.

In the realm of microelectronics, superlubricity could be a game changer. The inherent friction between moving parts often results in wear and tear, degrading the performance and shortening devices’ lifespans. However, through the application of superlubricity, such wear can be dramatically reduced. This offers the tantalizing possibility of significantly extended device lifespans, coupled with decreased maintenance requirements. In economic terms, this could lead to substantial cost savings, both for manufacturers and consumers. In addition, the enhanced performance of these devices could open the door to more complex and sophisticated microelectronic systems, accelerating the advancement of technology in this sector [72,73].

In the field of biomedical devices, the benefits of superlubricity could be groundbreaking. In orthopedic applications, such as artificial joints, the reduction of friction and wear is essential to minimize implant failure and enhance patient mobility and comfort. Superlubric materials and coatings can be employed to reduce friction at the articulating surfaces of joint replacements, leading to improved wear resistance and longevity. Researchers have explored the use of self-lubricating materials, such as hydrogels and polymer composites, to achieve superlubric conditions in orthopedic applications. These materials can provide lubrication and reduce friction, thereby reducing wear debris and the potential for inflammation or implant failure [74,75,76].

In the field of medical robotics, superlubricity plays a crucial role in ensuring smooth and precise movements of robotic surgical instruments. By incorporating superlubric coatings or materials in the joints and bearings of surgical robots, friction can be minimized, enabling more accurate and dexterous movements during surgical procedures. This enhances the surgeon’s control and precision, improving patient outcomes and reducing the risk of tissue damage.

Superlubricity is also relevant in dental applications. The movement of orthodontic wires and brackets can cause friction and discomfort for patients. By applying superlubric coatings to orthodontic wires, the frictional forces can be minimized, resulting in smoother tooth movement and reduced patient discomfort. In the field of ophthalmology, superlubricity has the potential to enhance the performance of contact lenses. Friction between the lens and the eye’s surface can lead to discomfort and dryness. By incorporating superlubric coatings or materials, contact lenses can exhibit reduced friction and improved lubrication, resulting in enhanced comfort and a prolonged wear time.

Superlubricity could also have transformative implications for the automotive industry. A significant amount of energy in vehicles is lost due to friction within various components, such as engines, transmissions, and brakes. If superlubricity could be effectively implemented, the energy loss due to friction could be drastically reduced, thereby improving fuel efficiency. In the automotive industry, researchers have focused on developing advanced superlubric coatings for engine components to enhance fuel efficiency. This could revolutionize the automotive industry, leading to more efficient vehicles, significant cost savings, and substantial reductions in environmental impacts [77,78].

In the field of power generation, superlubricity can improve the efficiency of conventional power plants and enhance the performance of advanced energy conversion systems. For example, in gas turbines, reducing friction in the rotating components, such as turbine blades and bearings, can lead to improved power output and reduced fuel consumption. Researchers are exploring the use of advanced coatings, such as diamond-like carbon (DLC) and nanostructured materials, to achieve superlubric conditions in gas turbines, enabling higher efficiency and reduced emissions. In the field of renewable energy, superlubric materials and coatings have the potential to enhance the efficiency of wind turbines, hydroelectric generators, and solar panel tracking systems. By reducing friction in the mechanical components of these systems, such as bearings and gears, superlubricity can improve energy conversion and increase the overall system efficiency. Researchers are exploring the use of advanced superlubric materials, including nanocomposites and self-lubricating coatings, to optimize renewable energy technologies [79].

The implications of superlubricity also extend to the realm of magnetic storage devices. In hard disk drives, friction between the spinning disk and the read/write head can lead to wear and tear, reducing the lifespan of the device and potentially compromising data integrity. Superlubricity could dramatically reduce this wear and tear, resulting in extended device lifespans and enhanced data reliability. This could have significant implications for data centers and IT infrastructure, leading to increased efficiency, reliability, and cost savings [80,81,82].

Superlubricity also holds significant promise in the subtractive manufacturing sector. Cutting tools, used in a variety of machining operations, can suffer from friction-induced wear, reducing precision and increasing energy consumption. The implementation of superlubricity could drastically reduce this wear, leading to more precise manufacturing processes, decreased energy consumption, and an increased tool lifespan. This could lead to significant cost savings and enhanced product quality within the manufacturing sector [83].

In the field of precision manufacturing, superlubricity has shown significant potential in improving the performance of microelectromechanical systems (MEMS) and nanoelectromechanical systems (NEMS). Researchers have focused on developing novel superlubric coatings and materials to reduce friction-induced stiction and improve the reliability of these miniature devices [84,85].

In additive manufacturing, superlubric coatings can enhance the performance of 3D printing processes by reducing friction between the printed object and the printing bed or nozzle. This can improve the print quality, enable the use of a wider range of materials, and enhance the overall efficiency of the printing process.

The aerospace industry stands to significantly gain from superlubricity. Friction and resultant wear and tear in various aircraft components, from engines to control systems, can be significantly reduced. Researchers are exploring the use of advanced solid lubricant coatings on aircraft engines and landing gears. These coatings, such as molybdenum disulfide (MoS_2_) and tungsten disulfide (WS_2_), offer excellent low-friction properties and can withstand the harsh operating conditions in aerospace environments. By reducing friction and wear, these coatings can improve fuel efficiency and extend the lifespan of critical components, leading to cost savings and enhanced safety in the aerospace industry [86,87].

The marine and shipping industry could also be a significant beneficiary of superlubricity. The reduced friction could mean less wear on ship components, increasing their lifespan, reducing maintenance needs, and enhancing the overall efficiency of marine vehicles. This could lead to substantial fuel savings and reduced emissions, contributing to the environmental sustainability of the marine industry [88].

In the realm of nanotechnology, the implications of superlubricity are particularly profound. On this scale, friction can pose serious challenges to the effective functioning of nanoscale devices. Superlubricity could lead to new possibilities in the design and application of nanodevices, including nanorobots, nano-sensors, and nanogenerators [89,90]. Nano-positioning systems, crucial in various applications such as scanning probe microscopy, atomic force microscopy, and semiconductor manufacturing, have also benefited from superlubric advancements. Researchers have explored the use of novel superlubric materials and coatings to improve the precision and accuracy of nano-positioners [45,91].

Furthermore, in the field of quantum technologies, superlubricity can play a role in reducing unwanted vibrations and mechanical noise in sensitive quantum systems. By minimizing friction-induced disturbances, superlubric materials and coatings can contribute to the stability and coherence of quantum systems, enabling advancements in quantum computing, sensing, and communication [62,92].

Superlubricity is a groundbreaking area of study with potentially revolutionary implications across a multitude of fields. While much research and development work is needed to fully harness its potential, the prospects are indeed exciting. From microelectronics to large-scale mechanical systems, biomedical devices to the automotive industry, the potential applications of superlubricity are vast. As our understanding of this unique phenomenon continues to deepen, it is likely to spawn a host of innovations, driving forward technological progress and offering solutions to some of our most challenging problems. As we stand on the precipice of this exciting frontier in friction management, the future of superlubricity holds promise and intrigue, signaling a new era in science and technology.

## 7. Challenges and Future Directions

The field of superlubricity has made significant progress in recent years, with remarkable advancements in understanding the underlying mechanisms and developing techniques to achieve near-zero friction. However, several challenges remain that need to be addressed for the widespread application of superlubric systems. Additionally, exploring new directions and pushing the boundaries of superlubricity research will open up exciting possibilities for future advancements.

Superlubricity, despite its numerous advantages, also presents certain disadvantages that need to be considered. Firstly, superlubricity can be highly sensitive to environmental conditions, such as temperature and humidity. Even slight variations in these factors can disrupt the delicate lubricating film, leading to a loss of superlubric behavior. Additionally, superlubric materials often have a limited load-bearing capacity, which can restrict their application in scenarios involving high loads or intense mechanical stresses. Furthermore, superlubric surfaces are susceptible to contamination, and even small amounts of particles or impurities can disrupt the lubricating layer and increase friction.

Another challenge lies in the selection and compatibility of superlubric materials. Identifying suitable materials that are chemically compatible, stable, and can maintain long-term performance can be complex. Additionally, the understanding and characterization of superlubricity remain areas of ongoing research, with the underlying mechanisms and accurate measurement techniques still being refined. Lastly, in certain applications, the extremely low friction associated with superlubricity may lead to reduced energy dissipation, which can have implications for systems that rely on controlled friction for optimal performance. Careful consideration of these limitations is necessary when implementing superlubricity, as addressing these challenges will contribute to unlocking the full potential of superlubric materials and technologies.

### 7.1. Technical Challenges in Achieving and Maintaining Superlubricity

One of the key technical challenges is the precise control and maintenance of superlubric conditions. Achieving superlubricity often requires specific environmental conditions, such as humidity levels or the presence of certain gases, which can be difficult to control in practical applications. Maintaining ultra-low-friction conditions over extended periods and under varying operating conditions is crucial for practical applications. Research has explored the development of robust and wear-resistant coatings, the optimization of lubricant compositions, and the study of nanoscale wear mechanisms to address these challenges [63,69,93,94,95,96]. For example, Fan et al. [95] synthesized hydrogenated graphene coatings with self-passivating and self-healing effects. The coatings exhibited superior lubricating properties and a long service lifetime, even under high loads and sliding speeds in ambient conditions.

Another challenge lies in scaling up superlubricity from the nanoscale to the macroscale. While superlubric behavior has been well-established at the atomic and molecular levels, replicating these effects in larger systems presents unique difficulties. Maintaining atomically smooth surfaces and controlling friction at larger scales requires innovative surface engineering techniques and the development of coatings and materials that can withstand real-world conditions [97,98,99].

The transition from laboratory-scale demonstrations to large-scale industrial applications poses significant challenges. Scaling up superlubricity requires considerations such as manufacturing processes, cost-effectiveness, and compatibility with existing industrial infrastructure. Recent advancements in deposition techniques, surface engineering methods, and material synthesis have shown promise in overcoming these challenges and enabling the widespread implementation of superlubricity in industrial settings. Another aspect of scaling up is the integration of superlubricity into existing machinery and systems. Retrofitting or designing new components with superlubric properties can be complex, requiring compatibility with existing materials, geometries, and operating conditions.

Sustainability is another important aspect that needs to be considered in superlubricity research. While superlubricity offers potential energy and resource savings through reduced friction and wear, there are also concerns related to the environmental impact of the materials and processes involved. One area of concern is the use of certain lubricants and coatings that may contain environmentally harmful substances. The development and application of superlubric systems should consider the environmental impact, resource consumption, and long-term performance. Researchers are exploring sustainable lubricants, eco-friendly coatings, and self-healing materials to address these concerns and ensure that superlubric technologies contribute to a more sustainable future [69,98,100]. Another aspect is the lifecycle analysis of superlubric materials and coatings. Understanding the environmental footprint of these materials, from production to disposal, is essential for ensuring their overall sustainability.

Another challenge lies in understanding and controlling the complex interplay of factors that contribute to superlubricity. The intricate balance between atomic structures, surface interactions, environmental conditions, and material properties requires a comprehensive understanding to achieve reliable and consistent superlubric behavior. Recent research has focused on developing advanced computational models and simulations to gain insights into the mechanisms underlying superlubricity and guide experimental efforts [16,28]. For example, Wang et al. [28] introduced a smoothed molecular dynamics method that incorporates a mapping strategy between atoms and a background mesh, enabling efficient atomic simulations with larger feasible timesteps. The findings provided valuable insights into the role of interfacial dynamics in achieving and maintaining superlubricity.

Addressing these technical challenges and overcoming the barriers to achieving and maintaining superlubricity is crucial for unlocking its full potential across various fields. Continued research efforts, interdisciplinary collaborations, and the integration of advanced characterization techniques will drive further advancements in the field of superlubricity and pave the way for its practical applications in different industries.

### 7.2. Potential Research Directions and Technological Innovations

The field of superlubricity presents numerous exciting research directions and technological innovations for the future. These include:

*Advanced Materials:* Continued exploration of new materials and composites with tailored atomic structures and properties can unlock novel superlubric effects. For example, researchers are investigating the potential of 2D materials beyond graphene, such as transition metal dichalcogenides (TMDs) [101] and MXenes [8], for achieving superlubricity. These materials offer unique properties and atomic structures that can be harnessed to enhance superlubricity in various applications.

*Surface Engineering Techniques:* Advancements in surface texturing and patterning techniques offer exciting prospects for achieving superlubricity. Researchers are exploring innovative approaches, including laser surface texturing [64] and self-assembled monolayers [102], to create customized surface structures that minimize friction and wear. By tailoring surface topographies and chemistries, it becomes possible to achieve superlubric behavior in a wide range of materials and applications.

*Multiscale Modeling and Simulation:* The development of advanced computational models and simulations is vital for understanding the complex dynamics of superlubric systems. Researchers are leveraging multiscale modeling techniques to bridge the gap between atomic-level interactions and macroscopic behavior [15,28]. This enables the prediction and optimization of superlubricity in different environments and under varying operating conditions, facilitating the design of more efficient and reliable superlubric systems.

*Self-Healing and Adaptive Coatings:* Self-healing coatings that can repair wear and damage on the surface hold great promise for maintaining long-term superlubricity. Researchers are exploring the use of stimuli-responsive materials and coatings that can self-repair or adapt to changing environmental conditions. For example, self-healing polymers and coatings that can autonomously fill in worn regions or regenerate lubricating layers have been investigated for achieving sustained superlubricity [95,103].

*In Situ Characterization Techniques:* Advancements in in situ characterization techniques enable real-time observation and measurement of friction, wear, and surface interactions during sliding processes. Techniques such as atomic force microscopy and scanning electron microscopy, combined with tribological testing systems, provide valuable insights into the fundamental mechanisms of superlubricity. These techniques allow researchers to visualize and understand the behavior of superlubric systems under different conditions, leading to improved design and optimization strategies.

*Smart Lubrication Systems:* The integration of smart lubrication systems offers the potential for dynamic control and adjustment of lubricating properties based on real-time conditions. For example, Berman et al. [104] explored smart solid lubricants that can self-generate at sliding interfaces, improving the efficiency of moving mechanical systems. They demonstrated that a mixture of graphene and iron nanoparticles undergoes a tribochemical reaction under high contact pressures, resulting in the formation of onion-like carbon nanostructures. By understanding and manipulating these tribochemical interactions, smart solid lubricants can be designed to enhance the overall performance of mechanical systems by maintaining superlubricity.

*Bioinspired Approaches:* Nature often provides inspiration for innovative solutions, and bioinspired approaches hold promise for superlubricity research. Researchers are studying the lubrication mechanisms found in biological systems, such as synovial joints and cartilage [105,106,107], to develop biomimetic lubricants and coatings. By mimicking the unique molecular and structural properties of biological lubrication, it may be possible to achieve superlubric behavior in synthetic systems.

The challenges and future directions of superlubricity research involve addressing technical hurdles in achieving and maintaining superlubric behavior, scaling up the technology for industrial applications, addressing sustainability concerns, and exploring new research directions and technological innovations. With ongoing advancements in materials science, surface engineering, computational modeling, and characterization techniques, the potential for superlubricity to revolutionize various industries and pave the way for more efficient and sustainable systems is promising.

## 8. Conclusions

In conclusion, superlubricity has emerged as a promising field of research with a wide range of potential applications. The ability to achieve ultra-low friction and minimal wear between surfaces leads to opportunities for improving the efficiency, performance, and lifespan of various systems and devices. Throughout this review, we have explored the fundamental principles, materials, techniques, and applications of superlubricity, highlighting recent research progress and advancements.

Superlubricity is rooted in the understanding of atomic structures, surface interactions, and environmental factors. By manipulating these factors, researchers have demonstrated the achievement of superlubric conditions at both the nanoscale and the macroscale. The use of layered materials, such as graphite and hexagonal boron nitride, has shown remarkable superlubric behavior due to the lack of lattice matching. Nanoscale techniques, including the use of nanoparticles and controlling the chemical environment, have further expanded the possibilities of achieving superlubricity.

In terms of materials, graphite, diamond-like carbon, and various 2D materials have emerged as prominent superlubric materials. Their unique atomic structures and properties allow for reduced friction and wear. Recent research has also explored the development of advanced synthetic materials and composites with tailored properties to achieve superlubric behavior.

The applications of superlubricity are vast and span across different industries. In mechanical systems, superlubricity has the potential to improve the efficiency and durability of engines, turbines, bearings, and gears. It offers opportunities for energy savings, reduced emissions, and extended lifespans. In biomedical applications, superlubricity can enhance the performance of artificial joints, robotic surgical instruments, and drug-delivery systems, contributing to improved patient outcomes and treatment efficacy. Additionally, emerging applications in energy storage, micro- and nano-scale systems, space exploration, and beyond demonstrate the growing potential and versatility of superlubricity.

However, several challenges must be addressed to fully harness the benefits of superlubricity. Technical challenges include understanding and controlling the complex interplay of factors that contribute to superlubricity, ensuring the durability and stability of superlubric systems, and scaling up the technology for practical industrial applications. Additionally, sustainability concerns regarding the environmental impact of materials and processes call for the development of eco-friendly lubricants, lifecycle analysis of superlubric systems, and optimization of manufacturing processes.

Looking ahead, the future of superlubricity holds great promise. Advances in computational modeling, surface engineering techniques, material synthesis, and lubrication strategies will continue to drive progress in the field. Scaling up superlubricity for industrial applications, addressing the sustainability concerns, and exploring new research directions and technological innovations will be critical in realizing the full potential of superlubricity.

In summary, superlubricity represents a significant advancement in the field of tribology and offers immense potential for various applications. Through the manipulation of atomic structures, surface interactions, and environmental conditions, researchers have achieved ultra-low friction and minimal wear, leading to improved efficiency, performance, and durability in mechanical systems, energy conversion, biomedical applications, and emerging technologies. Despite the challenges and sustainability considerations, the ongoing research efforts and collaborative initiatives hold promise for further advancements and the realization of a more efficient, reliable, and sustainable future.

## Figures and Tables

**Table 1:** Summary of materials exhibiting superlubric behavior.

Material	Atomic Structure	Surface Smoothness	Environmental Adaptability
**Graphite**	Layered, hexagonal	Atomically smooth	Sensitive to moisture and oxidation
**Diamond-Like Carbon (DLC)**	Amorphous carbon	Smooth, hard surface	High adaptability, excellent chemical stability, and resistance to wear
**Molybdenum Disulfide (MoS_2_)**	Layered, trigonal	Atomically smooth	Relatively stable in various environmental conditions
**Boron Nitride (h-BN)**	Layered, hexagonal	Atomically smooth	Excellent thermal stability and chemical resistance
**Tungsten Disulfide (WS_2_)**	Layered, trigonal	Atomically smooth	Stable under a range of environmental conditions
**Nanocomposites**	Composite structure	Varies	Depends on the matrix and nanoparticles
**Hydrogels**	Crosslinked polymer networks	Can be tailored	Dependent on water content
**Liquid Metals**	Amorphous, liquid	Smooth	Sensitive to temperature

**Table 2:** Comparison of lubricants for achieving superlubricity.

Lubricant	Friction Coefficient	Operating Temperature Range	Environmental Impact
**Graphene Oxide**	Ultra-low	Wide (−200 °C to 800 °C)	Generally low
**Ionic Liquids**	Low to ultra-low	Varies (depending on formulation)	Moderate
**MoS_2_**	Ultra-low	Wide (−150 °C to 400 °C)	Generally low
**WS_2_**	Ultra-low	Wide (−150 °C to 400 °C)	Generally low
**BN (h-BN)**	Ultra-low	Wide (−200 °C to 800 °C)	Generally low
**DLC**	Ultra-low	Wide (−200 °C to 800 °C)	Generally low
**Liquid Metals**	Ultra-low	High	Generally low
**Self-Assembled Monolayers**	Low to ultra-low	Limited (room temperature)	Generally low
**Bio-based Lubricants**	Low	Wide	Generally low
**Perfluoropolyether (PFPE)**	Low	Wide	Moderate
**Polydimethylsiloxane (PDMS)**	Low	Wide	Generally low
**Mineral Oil**	Moderate	Wide	Moderate
**Greases**	Moderate	Wide	High
**Water**	High	Limited	Low

**Table 3:** Selected applications of superlubricity in various industries.

Industry	Application	Benefits and Advancements
**Automotive**	Engine components	Improved fuel efficiency and reduced emissions
	Transmission systems	Enhanced power transmission efficiency
	Braking systems	Decreased wear and improved braking performance
**Aerospace**	Aircraft engines	Increased fuel efficiency and extended component lifespan
	Landing gear	Reduced friction and wear for smoother operation
	Satellite mechanisms	Enhanced reliability and functionality in space missions
**Manufacturing**	Precision machining	Reduced friction-induced errors and improved accuracy
	Micro- and nano-machining	Enhanced performance of MEMS and NEMS devices
**Energy**	Wind turbines	Increased energy conversion efficiency and extended lifespan
	Hydroelectric generators	Improved energy generation efficiency and reduced maintenance
	Battery systems	Minimized energy losses and improved charging/discharging rates
**Biomedical**	Orthopedic implants	Reduced friction and wear for longer implant lifespan
	Surgical robotics	Improved precision and control during surgical procedures
	Drug-delivery systems	Enhanced drug release and targeting efficacy
**Emerging Technologies**	Energy storage	Improved battery efficiency and performance
	Microfluidics	Reduced flow resistance and enhanced fluidic control
	Additive manufacturing	Minimized friction-induced defects and improved print quality
	Quantum technologies	Enhanced control and manipulation of quantum systems
